# Allopurinol Is an Independent Determinant of Improved Arterial Stiffness in Chronic Kidney Disease: A Cross-Sectional Study

**DOI:** 10.1371/journal.pone.0091961

**Published:** 2014-03-14

**Authors:** Khai P. Ng, Stephanie J. Stringer, Mark D. Jesky, Punit Yadav, Rajbir Athwal, Mary Dutton, Charles J. Ferro, Paul Cockwell

**Affiliations:** 1 Department of Renal Medicine, Queen Elizabeth Hospital Birmingham, Birmingham, United Kingdom; 2 School of Immunity and Infection, University of Birmingham, Birmingham, United Kingdom; University of Valencia, Spain

## Abstract

**Background:**

Arterial stiffness is increased in patients with CKD and is a powerful predictor of cardiovascular morbidity and mortality. Use of the xanthine oxidase inhibitor allopurinol has been shown to improve endothelial function, reduce left ventricular hypertrophy and possibly improve cardiovascular outcome. We explored the relationship between use of allopurinol and arterial stiffness in patients with chronic kidney disease (CKD).

**Methods:**

Cross-sectional observational study of 422 patients with CKD with evidence of, or at high risk of, renal disease progression. Arterial stiffness was determined by carotid-femoral pulse wave velocity (PWV).

**Results:**

The mean age was 63±16 years, median estimated glomerular filtration rate was 25 (interquartile range: 19–31) ml/min/1.73 m^2^ and mean PWV was 10.2±2.4 m/s. Seventy-seven patients (18%) were receiving regular allopurinol, 61% at a dose of 100 mg/day (range: 50–400 mg/day). Patients receiving allopurinol had significantly lower peripheral pulse pressure, central pulse pressure, central systolic blood pressure, serum uric acid level tissue advanced glycation end product levels but comparable high-sensitivity C-reactive protein levels. Use of allopurinol was associated with lower PWV. After adjusting for age, gender, ethnicity, tissue advanced glycation end product level, peripheral pulse pressure, smoking pack years, presence of diabetes mellitus and use of angiotensin converting enzyme inhibitor or angiotensin II receptor blocker, the use of allopurinol remained a significant independent determinant of PWV (mean difference: −0.63 m/s; 95% CI, −0.09 to −1.17 m/s, p = 0.02).

**Conclusion:**

In patients with CKD, use of allopurinol is independently associated with lower arterial stiffness. This study provides further justification for a large definitive randomised controlled trial examining the therapeutic potential of allopurinol to reduce cardiovascular risk in people with CKD.

## Introduction

Asymptomatic hyperuricaemia is associated with increased cardiovascular (CV) and all-cause mortality in the general population [1]–[6] and in patients with chronic kidney disease (CKD) [7]–[12]. Use of the xanthine oxidase inhibitor allopurinol in patients with CKD is associated with improvements in surrogate markers for CV disease (CVD) including endothelial function [13], [14] and left ventricular hypertrophy [14]. Furthermore, allopurinol use is associated with a slower progression of renal dysfunction [15] and a reduction in CV events [15], [16] in CKD cohorts.

Arterial stiffness is increased in patients with CKD and is a powerful predictor of mortality in this patient group [17]–[19]. Arterial stiffness is associated with endothelial dysfunction and increased left ventricular mass and is thought to be a key initiating factor contributing to the elevated CV risk observed in patients with CKD [17]. Carotid-femoral pulse wave velocity (PWV) is considered to be the current ‘gold-standard’ measurement of arterial stiffness [20]. Although allopurinol has been shown to improve endothelial function, lower central aortic pressure and regress left ventricular hypertrophy in patients with CKD, its effects on PWV remain unclear [14]. We therefore examined the relationship between allopurinol use and carotid-femoral PWV in patients with CKD recruited into the Renal Impairment In Secondary Care (RIISC) cohort study.

## Methods

### Ethics Statement

The study was approved by the South Birmingham Local Research Ethics committee (reference: 10/H1207/6) and all participants gave informed and written consent. The study was conducted in accordance with the Declaration of Helsinki. (Clinical Trials Registration Number: NCT01722383; Date of Registration: November 11, 2012)

### Study Design and Participants

The RIISC study is a prospective, observational cohort study of patients with CKD with evidence of, or at high risk of, renal disease progression. The inclusion and exclusion criteria have previously been reported in detail [21]. In brief, patients were included if they had stage 3 CKD with a declining estimated glomerular filtration rate (eGFR) of ≥5 ml/min/year or ≥10 ml/min/5years or a urine albumin creatinine ratio (uACR) ≥70 mg/mmol on three consecutive occasions, or stage 4/5 CKD. GFR was estimated (eGFR) using the four-variable Modification of Diet in Renal Disease (MDRD) equation with serum creatinine recalibrated to be traceable to an isotope derived mass spectroscopy method [22]. Patients with established renal failure receiving dialysis treatment and patients receiving immunosuppressive medication were excluded from the study. From October 2010 to November 2012, 437 out-patients under regular follow-up were recruited from renal clinics at two large teaching hospitals in the United Kingdom.

### Baseline measurements

Baseline clinical information on participants' demographics, renal diagnosis, diagnosis of diabetes mellitus (DM), CV history, past medication history, family history, concomitant medication, smoking and alcohol consumption history were recorded. Presence of CVD was defined by history or other evidence of angina, previous myocardial infarction, previous stroke or transient ischaemic attack, peripheral vascular disease, a previous revascularisation procedure or heart failure. Presence of DM was defined as receiving treatment for DM or a confirmed clinical diagnosis of diet-controlled DM. Smoking history and pack years were determined by participant self-reporting. An allopurinol user was defined as a participant who was receiving any dosage of allopurinol on recruitment. We contacted the primary care physician of all allopurinol users to obtain further details on the reason for prescription, presence of side effect related to allopurinol in the initial 3 months of treatment and start date of allopurinol to determine the duration of exposure.

Peripheral blood pressure (BP) was measured in the dominant arm using a British Hypertension Society approved automated oscillometric sphygmomanometer (BPM-100, BpTRU™), which obtained a series of six BP readings at 1-minute intervals after 5 minutes of rest [23]. Mean peripheral BP was derived from the average of the 2^nd^ to 6^th^ BP readings. Carotid-femoral PWV was measured non-invasively using the Vicorder system (Skidmore Medical, Bristol, UK) as previously described [24]. This is an operator independent and highly reproducible technique with low within-subject variation [24]. After 5 minutes of lying supine, PWV measurements were obtained in duplicate; the mean of the measurements was used in data analyses. Central pressure waveforms were derived and analysed using pulse wave analysis as previously described [25]. The central pressure waveform was analysed to determine the augmentation index (AIx) and central aortic pressures. AIx represents the difference between the second and first peaks of the central pressure waveform in systole, expressed as a percentage of the pulse pressure. Given the known effects of heart rate, AIx was corrected to a heart rate of 75 beats per minute (AIx_75_) [26].

Routine laboratory testing included blood haematological (Beckman Coulter Haematology Analyzer) and biochemical profiles and urinary albumin creatinine ratios (ACR) (Roche Hitachi 702 Analyser). Additional samples were centrifuged and serum was aliquoted and stored at −80°C and subsequently batch analysed for high sensitivity C-reactive protein (hsCRP) using a commercially available assay (SpaPlus assay, Binding Site). Tissue advanced glycation end product (AGE) level was determined by skin autofluorescence (SAF) using a validated AGE Reader™ (DiagnOptics BV, Groningen, The Netherlands).

### Statistical Analysis

Statistical analysis was performed using SPSS version 19.0 (SPSS Inc, Chicago, IL). Numerical values are expressed as mean ± standard deviation for parametric data or median (interquartile range) for non-parametric data. Normality of the distribution of data was assessed by visual inspection of histogram and normal probability plot [27]. Non-parametric variables were log transformed prior to analysis to achieve normal distribution. If normal distribution was not achieved after transformation, non-parametric tests were used. Parametric continuous data were compared using student t-tests and non-parametric using Mann-Whitney tests. Pearson or Spearman's bivariate correlation analysis was used to examine the relationship between parametric and non-parametric numerical variables, respectively. Correlation coefficient factors were expressed as ‘r’ for Pearson correlation analyses and ‘rho’ for Spearman's analysis. Categorical data were compared by χ^2^ tests. As age is strongly correlated with arterial stiffness, we divided the studied population into 4 age quartiles. Two-way analysis of variance (ANOVA) was performed to examine the interaction between age and the use of allopurinol as well as their individual effect on PWV. In addition, multiple linear regression was performed to explore the relationship between PWV and independent variables. Missing data was excluded by cases pairwise during analyses. Statistical significance is defined as a two-tailed p value <0.05.

## Results

### Baseline characteristics

**Figure 1:**
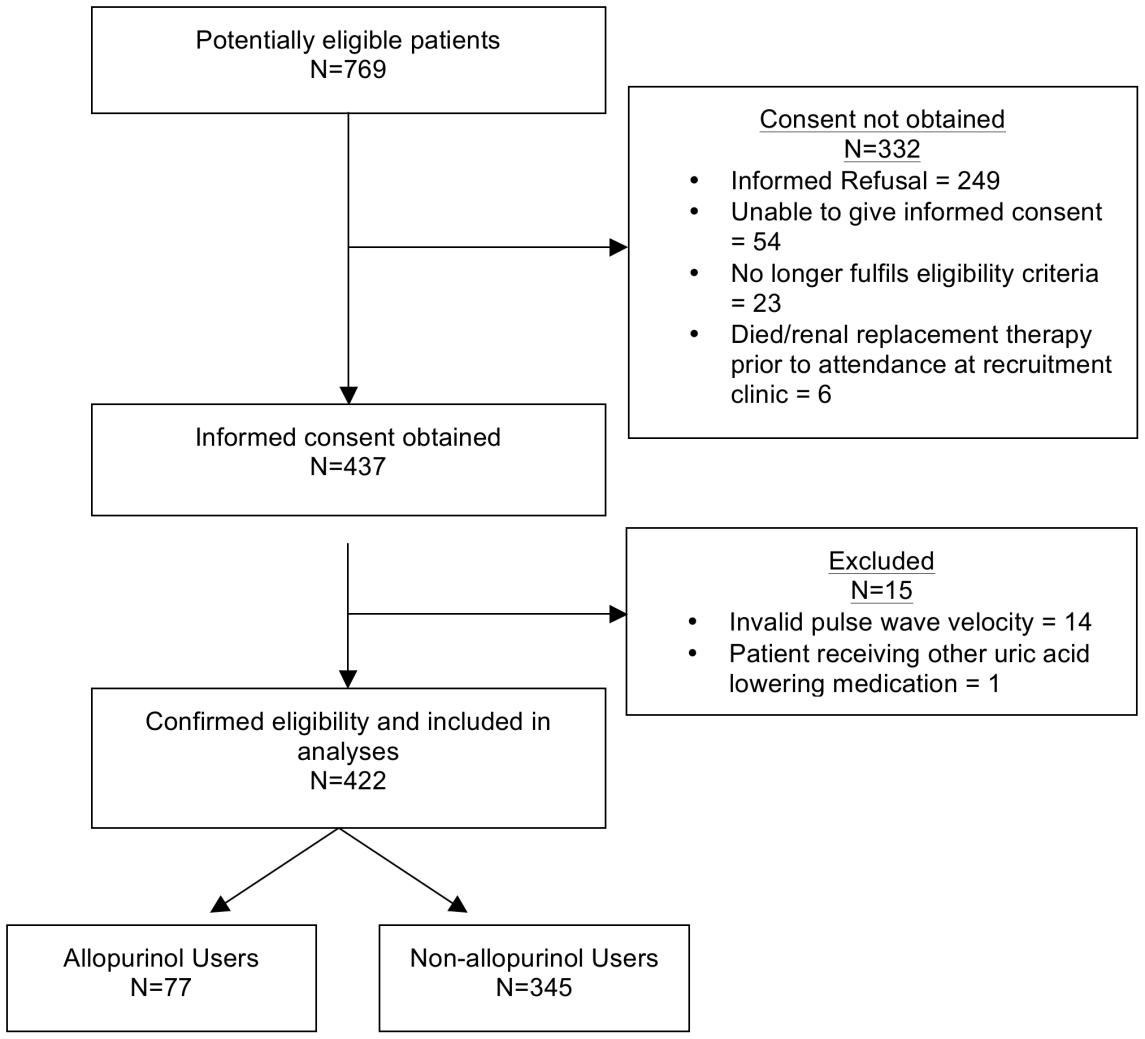
Flow diagram of the participants included in the study.

Four hundred and thirty-seven patients were recruited; of whom 14 did not have PWV measured for technical reasons and were therefore excluded from the study. One patient who was receiving febuxostat was excluded. Therefore 422 patients were included in the analyses. The numbers of individuals at each stage of study are detailed in Figure 1. The baseline demographic and biochemical characteristics of the study population are shown in Table 1. The mean age was 63±16 years with 60% of male gender and 71% of white ethnicity. Use of antihypertensive agents was common and 67% were receiving either an angiotensin converting enzyme inhibitor (ACEI) or angiotensin II receptor blocker (ARB). A small number (5%) were on both an ACEI and an ARB. There was a high prevalence of hyperuricaemia; 84% had a serum uric acid concentration greater than 360 µmol/L. The frequencies of different stages of CKD were: stage 1, 0.2%; stage 2, 1%; stage 3a, 5.3%; stage 3b, 23.1%; stage 4, 61.7%; stage 5, 8.7%. Seventy-seven patients (18%) were receiving regular allopurinol, 61% as a dose of 100 mg/day (range: 50–400 mg/day). Haemodynamic parameters are presented in Table 2.

**Table 1:** Baseline demographic, clinical and biochemical characteristic of all participants and according to the usage of allopurinol.

	All participants (n = 422)	Allopurinol user (n = 77)	Non-allopurinol user (n = 345)	P Value	Number missing data (%)
Age (years)	63±16	62±15	64±17	0.31	0
Male	225 (60%)	59 (77%)	196 (57%)	**0.001**	0
Ethnicity				**0.006**	1 (0.2)
White	301 (71%)	67 (87%)	234 (67%)		
Asian	64 (15%)	4 (5%)	60 (17%)		
Afro-Caribbean	40 (10%)	3 (4%)	37 (11%)		
Body Mass Index (kg/m^2^)	29.8±6.8	31.8±6.4	29.3±6.8	**0.003**	9 (2)
Presence of Diabetes Mellitus	152 (36%)	24 (31%)	128 (37%)	0.33	0
Presence of CVD	142 (34%)	21 (27%)	121 (35%)	0.19	0
Current smoker	58(14%)	4 (5%)	54 (16%)	**0.02**	0
Ex-smoker	174 (41%)	35 (46%)	139 (40%)	0.41	0
Smoking pack years *§§	2 (0–22)	0 (0–15)	2 (0–24)	0.25	13 (3)
Number of antihypertensive agents	2.4±1.3	2.5±1.0	2.4±1.4	0.49	6 (1)
Use of ACEI/ARB	281 (67%)	55 (71%)	227 (66%)	0.32	0
Use of thiazide	24 (6%)	1 (1%)	23 (7%)	0.10	0
Use of Antiplatelet agents	166 (39%)	37 (48%)	129 (37%)	0.08	0
Use of Statin	247 (59%)	46 (60%)	201 (58%)	0.81	0
Duration of allopurinol exposure	-	74±54	-	-	18 (23)
Serum creatinine*§ (µmol/L)	213 (169–263)	216 (174–270)	212 (167–263)	0.77	6 (1)
eGFR*§ (ml/min/1.73 m^2^)	25 (19–31)	26 (21–33)	24 (19–31)	0.24	6 (1)
Urine ACR*§ (mg/mmol)	35.0 (6.9–163.1)	40.1 (7.4–134.3)	33.8 (6.7–166.7)	0.70	45 (11)
Serum uric acid (µmol/L)	479±121	431±123	489±117	**<0.001**	8 (2)
Cholesterol (mmol/L)	4.6±1.2	4.5±1.1	4.7±1.2	0.33	5 (1)
Corrected calcium (mmol/L)	2.26±0.14	2.25±0.14	2.26±0.14	0.82	10 (2)
Phosphate*§ (mmol/L)	1.13 (0.99–1.28)	1.10 (1.00–1.23)	1.13 (0.98–1.30)	0.50	7 (1)
hsCRP*§ (mg/L)	3.280 (1.228–9.332)	3.678 (1.215–9.246)	3.203 (1.257–9.332)	0.92	103 (24)
SAF (a.u.)	3.0±0.8	2.8±0.7	3.1±0.8	**0.02**	60 (14)

Data are presented as frequency (percentage), mean ± standard deviation or *median (interquartile range).

*Parametric data was analysed using unpaired two-tailed t-test or Pearson's χ^2^ unless otherwise specified*.

§ *Log-transformed prior to analyses*.

§§ *Analysed using Mann-Whiteney U test*.

*Abbreviations: ACR = albumin creatinine ratio; bpm = beats per minutes; CVD = cardiovascular disease; ACEI = angiotensin converting enzyme inhibitor; ARB = Angiotensin II receptor blocker; eGFR = estimate glomerular filtration rate, hsCRP = high sensitivity C-reactive protein; SAF = skin autofluorescence*.

**Table 2:** Haemodynamic parameters of all participants and according to the usage of allopurinol.

	All participants (n = 422)	Allopurinol user (n = 77)	Non-allopurinol user (n = 345)	P Value	Number missing data (%)
Peripheral SBP (mmHg)	129±20	126±22	129±20	0.19	14 (3)
Peripheral DBP (mmHg)	76±13	76±12	75±13	0.59	14(3)
Peripheral PP (mmHg)	71±18	67±18	72±17	**0.02**	16 (4)
Central SBP (mmHg)	141±20	136±22	142±20	**0.02**	16 (4)
Central PP (mmHg)	65±18	61±18	66±17	**0.02**	16 (4)
AIx (%)	21±9	20±9	21±9	0.45	12 (3)
AIx_75_ (%)	21±9	20±8	21±9	0.35	12 (3)
Heart Rate (bpm)	69±13	68±15	69±15	0.42	8 (2)
PWV (m/s)	10.2±2.4	9.5±2.3	10.3±2.4	**0.006**	0

*Abbreviations: AIx: augmentation index; AIx_75_ = augmentation index adjusted to heart rate of 75 bpm; bpm = beats per minute; DBP = diastolic blood pressure; SBP = systolic blood pressure; PP = pulse pressure; PWV = pulse wave velocity*.

### Use of allopurinol

The demographic, clinical and biochemical characteristics of the cohort and a comparison between allopurinol users and non-allopurinol users is shown in Table 1. There was a significantly higher proportion of patients of male gender and white ethnicity and a significantly lower proportion of patients who were current smokers among allopurinol users. Allopurinol users had a higher body mass index (BMI) than non-allopurinol users. There was no significant difference in age, prevalence of DM, prevalence of CVD, percentage of ex-smokers, smoking pack years, total number of antihypertensive agents used and use of ACEI/ARB between the groups. Allopurinol users had significantly lower serum uric acid concentrations and lower SAF level compared to non-allopurinol users. Other biochemical variables, including kidney function, albuminuria, lipid and bone profiles and hsCRP levels were not different between the groups. Allopurinol users had significantly lower peripheral and central pulse pressures (PP), central systolic BP (SBP) and PWV (Table 2). There were no differences in heart rate, peripheral SBP, AIx and AIx_75_ between allopurinol and non-allopurinol users.

Among the 77 allopurinol users, details regarding allopurinol prescription were available from their primary care physician on 59 patients. Ninety four per cent were commenced on allopurinol for gout and 6% for asymptomatic hyperuricaemia. Side effects were reported in 12% during the first 3 months of allopurinol treatment: 3 had acute gout, 2 had a skin rash, 1 had diarrhoea and 1 complained of increased thirst. The mean duration of allopurinol use at recruitment was 74 months (SD: 54 months).

### Use of allopurinol and pulse wave velocity

Univariate correlations with PWV are shown in Table 3. Although BMI positively correlated with serum uric acid level (r = 0.178, p<0.001), there was no significant correlation between BMI and PWV. Uric acid levels, kidney function and hsCRP also did not correlate with PWV. In participants who were not receiving ACEI/ARB (n = 141), there was no correlation between uric acid and PWV (p = 0.66). Six per cent of participants were receiving a thiazide, use of this drug did not correlate with levels of uric acid (p = 0.58) or PWV (p = 0.66).

**Table 3:** Univariate analyses with pulse wave velocity (PWV) as the dependent outcome variable.

	Correlation coefficient	P value
**Demographics**		
Age (years)	0.534	**<0.001**
Gender (Male)	0.088	0.07
Ethnicity		
White	0.105	**0.03**
Asian	0.014	0.77
Afro-Caribbean	−0.105	**0.03**
Body Mass Index (kg/m^2^)	−0.057	0.25
Presence of diabetes mellitus	0.082	0.09
Presence of cardiovascular disease	0.044	0.37
Current smoker	0.021	0.67
Ex-smoker	0.198	**<0.001**
Smoking Pack Years**	0.244	**<0.001**
**Haemodynamics**		
BpTRU Peripheral SBP (mmHg)	0.320	**<0.001**
BpTRU Peripheral DBP (mmHg)	−0.061	0.21
Peripheral PP (mmHg)	0.454	**<0.001**
Central SBP (mmHg)	0.440	**<0.001**
Central PP (mmHg)	0.442	**<0.001**
AIx (%)	0.024	0.63
AIx_75_ (%)	0.023	0.64
**Biochemical markers**		
Serum creatinine* (µmol/L)	0.039	0.43
eGFR (ml/min/1.73 m^2^)*	−0.078	0.11
Urine ACR (mg/mmol)*	−0.042	0.40
Serum uric acid (µmol/L)	−0.035	0.48
Cholesterol (mmol/L)	−0.080	0.11
Corrected calcium (mmol/L)	−0.001	0.98
Phosphate (mmol/L)*	0.019	0.71
hsCRP (mg/L)*	0.062	0.27
SAF (a.u.)	0.253	**<0.001**
**Medications**		
Use of allopurinol	−0.135	**0.006**
Dose of allopurinol (mg)	−0.185	0.11
Duration of allopurinol exposure (months)	−0.019	0.89
Use of ACEI/ARB	−0.163	**0.001**
Use of thiazide	0.021	0.66

*Abbreviations: ACEI = angiotensin converting enzyme inhibitor; ACR = albumin creatinine ratio; AIx: augmentation index; AIx_75_ = augmentation index adjusted to heart rate of 75 bpm; ARB = angiotensin II receptor blocker; SBP = systolic blood pressure; DBP = diastolic blood pressure; PP = pulse pressure; SAF = skin autofluorescence*.

*Parametric data was analysed using Pearson correlation unless otherwise specified*.

*Natural Log transformed prior to analyses.

**Non-parametric data was analysed using Spearman's bivariate correlation analysis.

Pulse wave velocity positively correlated with increasing age, white ethnicity, SAF, peripheral and central SBP and PP. Ex-smokers and smoking pack years had a significant positive correlation with PWV whilst current smoking did not. Use of allopurinol (mean difference: −0.8 m/s; 95% CI, −0.2 to −1.4 m/s, p = 0.006), use of ACEI/ARB and Afro-Caribbean ethnicity were also associated with lower PWV. Neither the dose of allopurinol or duration of use of allopurinol had a significant correlation with PWV. Fifty-one per cent (n = 39) of the allopurinol users had a uric acid level below 416 µmol/L. Among allopurinol users, there was no difference in PWV between those with a uric acid below or above this threshold (p = 0.92).

**Figure 2:**
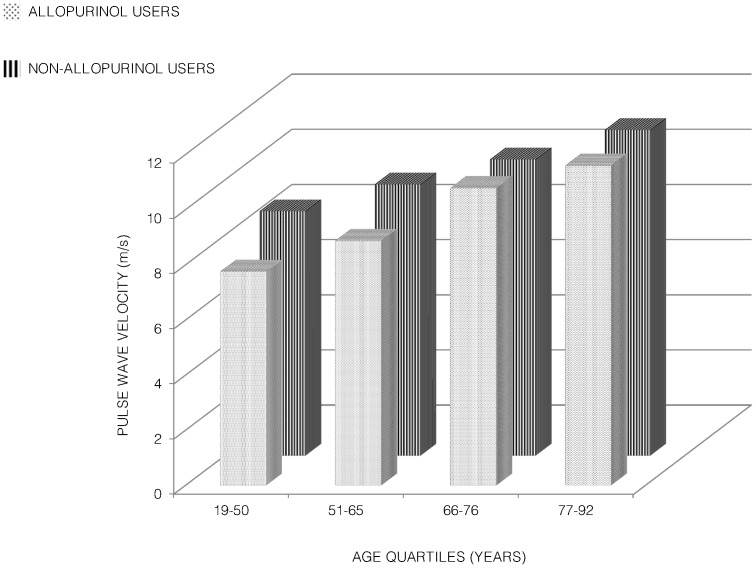
Differences in pulse wave velocity according to use of allopurinol and age quartiles.

Two-way ANOVA was used to explore the impact of age and use of allopurinol on PWV. Participants were divided into quartiles of age (19–50, 51–65, 66–76 and 77–92 years). Pulse wave velocity increased with age and was significantly lower in non-allopurinol users (Figure 2). There was no interaction between age and use of allopurinol (p = 0.27). There were significant main effects for both age and use of allopurinol, with age having a large effect size (partial eta squared = 0.201, p<0.001) and use of allopurinol having a small albeit significant effect size (partial eta squared = 0.011, p = 0.03).

A linear regression model was created with PWV as the dependent variable. Variables which correlated with PWV at a p value <0.1 were included in a standard regression model. As there was strong co-linearity among the BP measures, peripheral PP was selected from these parameters for incorporation in the regression model as it had the strongest correlation (r = 0.45, p<0.001) with PWV. Similarly, smoking pack years was selected to adjust for the relationship between smoking history and PWV in the regression model. Preliminary analyses were performed to ensure no violation of the assumptions of normality, linearity and multi-collinearity. Factors entered into the model were age, gender, ethnicity, smoking pack years, diagnosis of DM, SAF level, peripheral PP, use of ACEI/ARB and use of allopurinol. Age, peripheral PP and use of allopurinol were significant independent determinants of PWV (Table 4). In the regression model, the use of allopurinol was associated with a mean reduction of PWV of 0.63 m/s (95% CI, −0.09 to −1.17 m/s, p = 0.02). The model explained 35% of the variance in PWV. Substituting peripheral SBP, central SBP or central PP, for peripheral PP and substituting smoking pack years for current or previous smoking status made no appreciable difference to the model.

**Table 4:** Multiple regression with pulse wave velocity as the dependent outcome variable.

	Mean change of PWV	95% CI		P value
		Lower bound	Upper bound	
**Age (/5 years)**	**0.306**	**0.225**	**0.387**	**<0.001**
Gender (male)	0.423	−0.009	0.856	0.06
White ethnicity	0.252	−0.296	0.800	0.4
Afro-caribbean ethnicity	0.552	−0.245	1.350	0.2
Smoking pack years	0.005	−0.006	0.015	0.4
Presence of diabetes mellitus	0.163	−0.261	0.586	0.5
SAF (/1 a.u.)	−0.258	−0.559	0.043	0.09
**Peripheral PP (/5 mmHg)**	**0.186**	**−0.0121**	**0.251**	**<0.001**
Use of ACEI/ARB	−0.136	−0.579	0.307	0.5
**Use of allopurinol**	**−0.633**	**−1.174**	**−0.092**	**0.02**

*Adjusted R^2^ for model = 0.348, p<0.001*.

*Abbreviations: ACEI = angiotensin converting enzyme inhibitor; ARB = angiotensin II receptor blocker; CI = confidence interval; PP = pulse pressure, PWV = pulse wave velocity; SAF = skin autofluorescence*.

## Discussion

This observational study in a prospectively recruited CKD cohort suggests that the use of allopurinol was associated with reduced arterial stiffness as measured by carotid-femoral PWV, the current gold-standard measurement of arterial stiffness [20]; this association was independent of age and BP. Arterial stiffness, which is a hallmark of CKD, is a well-recognised, powerful prognostic marker of CV morbidity and mortality in both the general and CKD population [17]–[19]. Increased arterial stiffness results in higher systolic pressures, greater pressure fluctuations and leads to ventricular-arterial uncoupling, myocardial hypertrophy and fibrosis. Alterations in the extracellular matrix and endothelial dysfunction promoted by chronic inflammation, increase oxidative stress and accumulation of advanced glycation end products, vascular calcification, and activation of renin-angiotensin-aldosterone system (RAAS) have been postulated to contribute to increased arterial stiffness [17], [28].

Allopurinol, a xanthine oxidase inhibitor, is commonly prescribed for patients with gout as a uric acid lowering agent. During the catalytic reaction that produces uric acid, xanthine oxidase generates reactive oxidative species, which may contribute to the development of endothelial dysfunction, hypertension and vascular damage [29]. Accumulating evidence from interventional studies indicates that allopurinol improves endothelial dysfunction [14], [30], lowers left ventricular mass [14], and may slow progression of CKD and lower CV risk [15]. The results of this current study suggest that some of the beneficial effect of allopurinol may occur through reducing arterial stiffness.

Even among allopurinol users there was a high prevalence of hyperuricaemia. Although several large observational studies have reported a strong association between hyperuricaemia and CV morbidity or mortality, the evidence for hyperuricaemia as a risk factor or risk marker of CVD is conflicting [31]–[39]. Despite a significant association between allopurinol use and PWV, we found no significant direct association between serum uric acid levels and PWV. In a small group of patients with chronic heart failure, George et al demonstrated that the mechanism of improvement in endothelial function with allopurinol was attributable to reduced oxidative stress and not to uric acid reduction [30]. CKD is known to be associated with increased oxidative stress and acute-phase inflammation, which may both contribute to increased CV risk [40]. We found no significant difference in the levels of hsCRP between allopurinol users and non-allopurinol users, suggesting that inflammation was not a prominent mechanism in this association. Advanced glycation end product (AGE) has a bi-directional relationship with oxidative stress, including studies showing that increased oxidative stress is associated with formation and accumulation of AGE [41]–[45]. Level of tissue AGE as measured by SAF, which positively correlated with arterial stiffness, was found to be significantly lower in the allopurinol users when compared to non-allopurinol users, indicating that this biological pathway may be relevant to the link between allopurinol use and PWV described here.

As arterial stiffness and BP are closely related, it is possible that the effect of allopurinol on arterial stiffness may be mediated through improved BP. A recent meta-analysis showed that allopurinol is associated with a small but significant reduction in BP [46]. Several hypotheses have been postulated to explain this apparent association. The antioxidant effect of allopurinol was considered to play a major role in improving endothelial function and blood pressure regulation [46]. Nonetheless, there was no clear consensus to-date with regards to the effects of oral antioxidant on arterial blood pressure. While some demonstrated blood pressure lowering effect of antioxidant vitamins [47], [48], others did not [49]–[52] and one study showed paradoxical blunting of exercise training-induced improvement in endothelial function with antioxidant administration [53]. In addition to its antioxidant property, there is emerging evidence that allopurinol can block the deleterious cardiovascular effect of angiotensin II [54]–[56]. In this current study we found a significant difference in both peripheral and central BP between allopurinol users and non-allopurinol users, despite a comparable prevalence of ACEI/ARB use and total numbers of anti-hypertensive agents between groups. However, after adjustment in a multivariate analysis, the use of allopurinol remained significantly associated with arterial stiffness. This suggests a distinct direct effect of allopurinol on vascular stiffness that is independent of BP and the use of ACEI/ARB. This observation is supported by an RCT of 66 patients with mild to moderate hypertension which reported a favourable effect of allopurinol on aortic compliance, independent of ACEI or thiazide-based antihypertensive therapy [57].

### Limitations

There were a number of limitations in this study. Due to the observational, cross-sectional nature of the study and non-randomised use of allopurinol, the association between allopurinol use and lower arterial stiffness reported here does not prove causality.

Although there were unequal distributions of gender, race, BMI, current smoking status and differences in serum uric acid between the groups, these are unlikely to have resulted in bias. Male gender was associated with higher PWV, however despite a higher proportion of males amongst allopurinol users, use of allopurinol remained associated with a lower PWV. In addition, people of Afro-Caribbean ethnicity had a lower PWV; most of the Afro-Caribbean participants were non-allopurinol users. The unequal distribution of gender and race between the groups was therefore unlikely to have resulted in bias against non-allopurinol use. As the number of non-white participants was small, we were unable to confidently examine the influence of allopurinol in different ethnic groups; this should be an area for future study. Although there were differences in BMI and serum uric acid level between the groups, these parameters did not have a significant bivariate association with PWV; hence, they were unlikely to confound the findings. Smoking history is known to have significant influence on arterial stiffness and there was a higher proportion of current smokers in the allopurinol non-user group. In addition, the comparatively lower peripheral and central pressures in the allopurinol user group might have contributed to lower PWV as BP is a strong determinant of arterial stiffness. However, after adjusted for haemodynamic parameters and smoking history in the regression model, use of allopurinol remained associated with lower PWV.

Although all available confounding variables were included in this study, there may be other potential unknown confounders as the biology of vascular disease in CKD is complex. The measurement of hsCRP was performed only at single time-point rather than the two time-points two weeks apart recommended by the American Heart Association [58]. We did not have measurements of endothelial dysfunction, which is closely linked to arterial stiffness and CKD [17]. Finally, encouraging results have been reported on an effect of allopurinol in improving renal function in patients with asymptomatic hyperuricaemia [59] or delaying disease progression in patients with CKD [15], [60]. However, due to the cross-sectional nature of the data we were unable to examine this relationship.

## Conclusion

In summary, the data shown here suggests that allopurinol is independently associated with lower arterial stiffness in patients with progressive CKD. This adds to accumulating evidence of the favourable effect of allopurinol on cardiovascular outcomes in a well-defined CKD cohort and indicates one mechanism by which this may occur. This study provides further justification for a large definitive RCT examining the therapeutic potential of allopurinol to reduce cardiovascular risk in people with CKD.

## References

[1] Perlstein TS, Gumieniak O, Williams GH, Sparrow D, Vokonas PS, et al (2006) Uric acid and the development of hypertension: the normative aging study. Hypertension 48: 1031–1036. 17060508 10.1161/01.HYP.0000248752.08807.4c

[2] Erdogan D, Gullu H, Caliskan M, Yildirim E, Bilgi M, et al (2005) Relationship of serum uric acid to measures of endothelial function and atherosclerosis in healthy adults. International journal of clinical practice 59: 1276–1282. 16236080 10.1111/j.1742-1241.2005.00621.x

[3] Freedman DS, Williamson DF, Gunter EW, Byers T (1995) Relation of serum uric acid to mortality and ischemic heart disease. The NHANES I Epidemiologic Follow-up Study. American journal of epidemiology 141: 637–644. 7702038 10.1093/oxfordjournals.aje.a117479

[4] Fang J, Alderman MH (2000) Serum uric acid and cardiovascular mortality the NHANES I epidemiologic follow-up study, 1971–1992. National Health and Nutrition Examination Survey. JAMA : the journal of the American Medical Association 283: 2404–2410. 10815083 10.1001/jama.283.18.2404

[5] Strasak A, Ruttmann E, Brant L, Kelleher C, Klenk J, et al (2008) Serum uric acid and risk of cardiovascular mortality: a prospective long-term study of 83,683 Austrian men. Clinical chemistry 54: 273–284. 18039719 10.1373/clinchem.2007.094425

[6] Strasak AM, Kelleher CC, Brant LJ, Rapp K, Ruttmann E, et al (2008) Serum uric acid is an independent predictor for all major forms of cardiovascular death in 28,613 elderly women: a prospective 21-year follow-up study. International journal of cardiology 125: 232–239. 18237790 10.1016/j.ijcard.2007.11.094

[7] Edwards NL (2008) The role of hyperuricemia and gout in kidney and cardiovascular disease. Cleveland Clinic journal of medicine 75 Suppl 5: S13–16. 10.3949/ccjm.75.suppl_5.s13 18822470

[8] Chonchol M, Shlipak MG, Katz R, Sarnak MJ, Newman AB, et al (2007) Relationship of uric acid with progression of kidney disease. American journal of kidney diseases : the official journal of the National Kidney Foundation 50: 239–247. 17660025 10.1053/j.ajkd.2007.05.013

[9] Madero M, Sarnak MJ, Wang X, Greene T, Beck GJ, et al (2009) Uric acid and long-term outcomes in CKD. American journal of kidney diseases : the official journal of the National Kidney Foundation 53: 796–803. 19303683 10.1053/j.ajkd.2008.12.021 PMC2691553

[10] Iseki K, Ikemiya Y, Inoue T, Iseki C, Kinjo K, et al (2004) Significance of hyperuricemia as a risk factor for developing ESRD in a screened cohort. American journal of kidney diseases : the official journal of the National Kidney Foundation 44: 642–650. 15384015

[11] Syrjanen J, Mustonen J, Pasternack A (2000) Hypertriglyceridaemia and hyperuricaemia are risk factors for progression of IgA nephropathy. Nephrology, dialysis, transplantation : official publication of the European Dialysis and Transplant Association - European Renal Association 15: 34–42. 10.1093/ndt/15.1.34 10607765

[12] Li YL, Wang L, Li J, Huang Y, Yuan WM (2011) [The correlation between uric acid and the incidence and prognosis of kidney diseases: a systematic review and meta-analysis of cohort studies]. Zhonghua nei ke za zhi [Chinese journal of internal medicine] 50: 555–561. 22041264

[13] Yelken B, Caliskan Y, Gorgulu N, Altun I, Yilmaz A, et al (2012) Reduction of uric acid levels with allopurinol treatment improves endothelial function in patients with chronic kidney disease. Clinical nephrology 77: 275–282. 22445470 10.5414/cn107352

[14] Kao MP, Ang DS, Gandy SJ, Nadir MA, Houston JG, et al (2011) Allopurinol benefits left ventricular mass and endothelial dysfunction in chronic kidney disease. Journal of the American Society of Nephrology : JASN 22: 1382–1389. 21719783 10.1681/ASN.2010111185 PMC3137586

[15] Goicoechea M, de Vinuesa SG, Verdalles U, Ruiz-Caro C, Ampuero J, et al (2010) Effect of allopurinol in chronic kidney disease progression and cardiovascular risk. Clinical journal of the American Society of Nephrology : CJASN 5: 1388–1393. 20538833 10.2215/CJN.01580210 PMC2924417

[16] Wei L, Mackenzie IS, Chen Y, Struthers AD, MacDonald TM (2011) Impact of allopurinol use on urate concentration and cardiovascular outcome. British journal of clinical pharmacology 71: 600–607. 21395653 10.1111/j.1365-2125.2010.03887.x PMC3080649

[17] Chue CD, Townend JN, Steeds RP, Ferro CJ (2010) Arterial stiffness in chronic kidney disease: causes and consequences. Heart 96: 817–823. 20406771 10.1136/hrt.2009.184879

[18] Vlachopoulos C, Aznaouridis K, Stefanadis C (2010) Prediction of cardiovascular events and all-cause mortality with arterial stiffness: a systematic review and meta-analysis. Journal of the American College of Cardiology 55: 1318–1327. 20338492 10.1016/j.jacc.2009.10.061

[19] Blacher J, Guerin AP, Pannier B, Marchais SJ, Safar ME, et al (1999) Impact of aortic stiffness on survival in end-stage renal disease. Circulation 99: 2434–2439. 10318666 10.1161/01.cir.99.18.2434

[20] Laurent S, Cockcroft J, Van Bortel L, Boutouyrie P, Giannattasio C, et al (2006) Expert consensus document on arterial stiffness: methodological issues and clinical applications. European heart journal 27: 2588–2605. 17000623 10.1093/eurheartj/ehl254

[21] Stringer S, Sharma P, Dutton M, Jesky M, Ng K, et al (2013) The natural history of, and risk factors for, progressive Chronic Kidney Disease (CKD): the Renal Impairment in Secondary care (RIISC) study; rationale and protocol. BMC nephrology 14: 95. 23617441 10.1186/1471-2369-14-95 PMC3664075

[22] Levey AS, Bosch JP, Lewis JB, Greene T, Rogers N, et al (1999) A more accurate method to estimate glomerular filtration rate from serum creatinine: a new prediction equation. Modification of Diet in Renal Disease Study Group. Annals of internal medicine 130: 461–470. 10075613 10.7326/0003-4819-130-6-199903160-00002

[23] Wright JM, Mattu GS, Perry TL Jr, Gelferc ME, Strange KD, et al (2001) Validation of a new algorithm for the BPM-100 electronic oscillometric office blood pressure monitor. Blood pressure monitoring 6: 161–165. 11518840 10.1097/00126097-200106000-00008

[24] Hickson SS, Butlin M, Broad J, Avolio AP, Wilkinson IB, et al (2009) Validity and repeatability of the Vicorder apparatus: a comparison with the SphygmoCor device. Hypertension research : official journal of the Japanese Society of Hypertension 32: 1079–1085. 19779487 10.1038/hr.2009.154

[25] Pucci G, Cheriyan J, Hubsch A, Hickson SS, Gajendragadkar PR, et al (2013) Evaluation of the Vicorder, a novel cuff-based device for the noninvasive estimation of central blood pressure. Journal of hypertension 31: 77–85. 23079681 10.1097/HJH.0b013e32835a8eb1

[26] Wilkinson IB, MacCallum H, Flint L, Cockcroft JR, Newby DE, et al (2000) The influence of heart rate on augmentation index and central arterial pressure in humans. The Journal of physiology 525 Pt 1: 263–270. 10811742 10.1111/j.1469-7793.2000.t01-1-00263.x PMC2269933

[27] Altman D (1999) Practical statistics for medical research. London: Chapman & Hall.

[28] Kals J, Kampus P, Kals M, Zilmer K, Kullisaar T, et al (2006) Impact of oxidative stress on arterial elasticity in patients with atherosclerosis. American journal of hypertension 19: 902–908. 16942931 10.1016/j.amjhyper.2006.02.003

[29] Touyz RM (2004) Reactive oxygen species, vascular oxidative stress, and redox signaling in hypertension: what is the clinical significance? Hypertension 44: 248–252. 15262903 10.1161/01.HYP.0000138070.47616.9d

[30] George J, Carr E, Davies J, Belch JJ, Struthers A (2006) High-dose allopurinol improves endothelial function by profoundly reducing vascular oxidative stress and not by lowering uric acid. Circulation 114: 2508–2516. 17130343 10.1161/CIRCULATIONAHA.106.651117

[31] Culleton BF, Larson MG, Kannel WB, Levy D (1999) Serum uric acid and risk for cardiovascular disease and death: the Framingham Heart Study. Annals of internal medicine 131: 7–13. 10391820 10.7326/0003-4819-131-1-199907060-00003

[32] Alderman MH, Cohen H, Madhavan S, Kivlighn S (1999) Serum uric acid and cardiovascular events in successfully treated hypertensive patients. Hypertension 34: 144–150. 10406838 10.1161/01.hyp.34.1.144

[33] Liese AD, Hense HW, Lowel H, Doring A, Tietze M, et al (1999) Association of serum uric acid with all-cause and cardiovascular disease mortality and incident myocardial infarction in the MONICA Augsburg cohort. World Health Organization Monitoring Trends and Determinants in Cardiovascular Diseases. Epidemiology 10: 391–397. 10401873 10.1097/00001648-199907000-00006

[34] Bos MJ, Koudstaal PJ, Hofman A, Witteman JC, Breteler MM (2006) Uric acid is a risk factor for myocardial infarction and stroke: the Rotterdam study. Stroke; a journal of cerebral circulation 37: 1503–1507. 10.1161/01.STR.0000221716.55088.d4 16675740

[35] Moriarity JT, Folsom AR, Iribarren C, Nieto FJ, Rosamond WD (2000) Serum uric acid and risk of coronary heart disease: Atherosclerosis Risk in Communities (ARIC) Study. Annals of epidemiology 10: 136–143. 10813506 10.1016/s1047-2797(99)00037-x

[36] Hozawa A, Folsom AR, Ibrahim H, Nieto FJ, Rosamond WD, et al (2006) Serum uric acid and risk of ischemic stroke: the ARIC Study. Atherosclerosis 187: 401–407. 16239005 10.1016/j.atherosclerosis.2005.09.020

[37] Niskanen LK, Laaksonen DE, Nyyssonen K, Alfthan G, Lakka HM, et al (2004) Uric acid level as a risk factor for cardiovascular and all-cause mortality in middle-aged men: a prospective cohort study. Archives of internal medicine 164: 1546–1551. 15277287 10.1001/archinte.164.14.1546

[38] Krishnan E, Baker JF, Furst DE, Schumacher HR (2006) Gout and the risk of acute myocardial infarction. Arthritis and rheumatism 54: 2688–2696. 16871533 10.1002/art.22014

[39] Verdecchia P, Schillaci G, Reboldi G, Santeusanio F, Porcellati C, et al (2000) Relation between serum uric acid and risk of cardiovascular disease in essential hypertension. The PIUMA study. Hypertension 36: 1072–1078. 11116127 10.1161/01.hyp.36.6.1072

[40] Oberg BP, McMenamin E, Lucas FL, McMonagle E, Morrow J, et al (2004) Increased prevalence of oxidant stress and inflammation in patients with moderate to severe chronic kidney disease. Kidney international 65: 1009–1016. 14871421 10.1111/j.1523-1755.2004.00465.x

[41] Schmidt AM, Hori O, Brett J, Yan SD, Wautier JL, et al (1994) Cellular receptors for advanced glycation end products. Implications for induction of oxidant stress and cellular dysfunction in the pathogenesis of vascular lesions. Arteriosclerosis and thrombosis : a journal of vascular biology/American Heart Association 14: 1521–1528. 10.1161/01.atv.14.10.1521 7918300

[42] Mulder DJ, de Boer JF, Graaff R, de Vries R, Annema W, et al (2011) Skin autofluorescence is inversely related to HDL anti-oxidative capacity in type 2 diabetes mellitus. Atherosclerosis 218: 102–106. 21665206 10.1016/j.atherosclerosis.2011.05.011

[43] Mulder DJ, van Haelst PL, Gross S, de Leeuw K, Bijzet J, et al (2008) Skin autofluorescence is elevated in patients with stable coronary artery disease and is associated with serum levels of neopterin and the soluble receptor for advanced glycation end products. Atherosclerosis 197: 217–223. 17499742 10.1016/j.atherosclerosis.2007.03.027

[44] Yan SD, Schmidt AM, Anderson GM, Zhang J, Brett J, et al (1994) Enhanced cellular oxidant stress by the interaction of advanced glycation end products with their receptors/binding proteins. The Journal of biological chemistry 269: 9889–9897. 8144582

[45] Yamagishi S, Maeda S, Matsui T, Ueda S, Fukami K, et al (2012) Role of advanced glycation end products (AGEs) and oxidative stress in vascular complications in diabetes. Biochimica et biophysica acta 1820: 663–671. 21440603 10.1016/j.bbagen.2011.03.014

[46] Agarwal V, Hans N, Messerli FH (2012) Effect of Allopurinol on Blood Pressure: A Systematic Review and Meta-Analysis. The Journal of Clinical Hypertension no–no. 10.1111/j.1751-7176.2012.00701.x PMC8033809 23730993

[47] Schutte AE, Huisman HW, Oosthuizen W, van Rooyen JM, Jerling JC (2004) Cardiovascular effects of oral Supplementation of vitamin C, E and folic acid in young healthy males. International journal for vitamin and nutrition research Internationale Zeitschrift fur Vitamin- und Ernahrungsforschung Journal international de vitaminologie et de nutrition 74: 285–293. 15580811 10.1024/0300-9831.74.4.285

[48] Galley HF, Thornton J, Howdle PD, Walker BE, Webster NR (1997) Combination oral antioxidant supplementation reduces blood pressure. Clinical science 92: 361–365. 9176034 10.1042/cs0920361

[49] Miller ER 3rd, Appel LJ, Levander OA, Levine DM (1997) The effect of antioxidant vitamin supplementation on traditional cardiovascular risk factors. Journal of cardiovascular risk 4: 19–24. 9215516 10.1177/174182679700400104

[50] Zureik M, Galan P, Bertrais S, Mennen L, Czernichow S, et al (2004) Effects of long-term daily low-dose supplementation with antioxidant vitamins and minerals on structure and function of large arteries. Arteriosclerosis, thrombosis, and vascular biology 24: 1485–1491. 10.1161/01.ATV.0000136648.62973.c8 15217803

[51] Eskurza I, Monahan KD, Robinson JA, Seals DR (2004) Ascorbic acid does not affect large elastic artery compliance or central blood pressure in young and older men. American journal of physiology Heart and circulatory physiology 286: H1528–1534. 15020306 10.1152/ajpheart.00879.2003

[52] Mottram P, Shige H, Nestel P (1999) Vitamin E improves arterial compliance in middle-aged men and women. Atherosclerosis 145: 399–404. 10488969 10.1016/s0021-9150(99)00073-8

[53] Wray DW, Uberoi A, Lawrenson L, Bailey DM, Richardson RS (2009) Oral antioxidants and cardiovascular health in the exercise-trained and untrained elderly: a radically different outcome. Clinical science 116: 433–441. 18795893 10.1042/CS20080337

[54] Jia N, Dong P, Ye Y, Qian C, Dai Q (2012) Allopurinol attenuates oxidative stress and cardiac fibrosis in angiotensin II-induced cardiac diastolic dysfunction. Cardiovascular therapeutics 30: 117–123. 20973927 10.1111/j.1755-5922.2010.00243.x

[55] Ercan ZS, Ilhan M, Oguz A, Turker RK (1992) Superoxide dismutase and allopurinol prevent the pressor effects of angiotensin II and histamine in the guinea-pig isolated perfused lung exposed to hypoxia. General pharmacology 23: 1149–1151. 1487126 10.1016/0306-3623(92)90303-2

[56] Kasal DA, Neves MF, Oigman W, Mandarim-de-Lacerda CA (2008) Allopurinol attenuates L-NAME induced cardiomyopathy comparable to blockade of angiotensin receptor. Histology and histopathology 23: 1241–1248. 18712676 10.14670/HH-23.1241

[57] Kostka-Jeziorny K, Uruski P, Tykarski A (2011) Effect of allopurinol on blood pressure and aortic compliance in hypertensive patients. Blood pressure 20: 104–110. 21405957 10.3109/08037051.2010.532323

[58] Pearson TA, Mensah GA, Alexander RW, Anderson JL, Cannon RO 3rd, et al (2003) Markers of inflammation and cardiovascular disease: application to clinical and public health practice: A statement for healthcare professionals from the Centers for Disease Control and Prevention and the American Heart Association. Circulation 107: 499–511. 12551878 10.1161/01.cir.0000052939.59093.45

[59] Kanbay M, Ozkara A, Selcoki Y, Isik B, Turgut F, et al (2007) Effect of treatment of hyperuricemia with allopurinol on blood pressure, creatinine clearence, and proteinuria in patients with normal renal functions. International urology and nephrology 39: 1227–1233. 17701281 10.1007/s11255-007-9253-3

[60] Siu Y-P, Leung K-T, Tong MK-H, Kwan T-H (2006) Use of Allopurinol in Slowing the Progression of Renal Disease Through Its Ability to Lower Serum Uric Acid Level. american journal of kidney diseases 47: 51–59. 16377385 10.1053/j.ajkd.2005.10.006

